# Case report: MRI findings with CNS blastomycosis in three domestic cats

**DOI:** 10.3389/fvets.2022.966853

**Published:** 2022-08-16

**Authors:** Silke Hecht, Jennifer R. Michaels, Heather Simon

**Affiliations:** ^1^Department of Small Animal Clinical Sciences, University of Tennessee, Knoxville, TN, United States; ^2^Department of Neurology and Neurosurgery, Angell Animal Medical Center, Boston, MA, United States

**Keywords:** feline, brain, central nervous system, fungal, encephalitis, magnetic resonance imaging, *Blastomyces dermatitidis*

## Abstract

Blastomycosis is a systemic mycotic infection caused by dimorphic fungi. The disease is rare in cats, and reports on imaging findings with central nervous system (CNS) involvement are limited. Magnetic resonance imaging (MRI) was performed antemortem in three feline patients. Imaging findings that may allow prioritization of intracranial blastomycosis over other differential diagnoses included focal or multifocal intra-axial mass lesions with dural contact, lesion hypointensity on T2-weighted images and diffusion-weighted imaging/apparent diffusion coefficient map (DWI/ADC), strong and homogeneous contrast enhancement of the lesion(s), concurrent meningeal enhancement, marked perilesional edema and mass-effect, and ocular abnormalities. One cat was managed successfully and had a recurrence of CNS blastomycosis more than 4.5 years after the initial diagnosis. Repeat MRI at that point revealed both new and persistent (chronic) abnormalities.

## Introduction

Blastomycosis is a systemic mycotic infection caused by dimorphic fungi. *Blastomyces dermatitidis* and *Blastomyces gilchristii* have an endemic distribution in the Mississippi, Missouri, and Ohio River valleys, the mid-Atlantic states, and some Canadian provinces with sporadic cases reported in other regions (1–3). Disease transmission occurs predominantly through inhalation, resulting in infection of the respiratory system. Other organ systems that may be involved include the regional lymph nodes, the skin, the urogenital system, the eyes, and the central nervous system (CNS) (4–9).

Unlike dogs, the disease is uncommon in domestic cats (2, 3, 10, 11). Clinical signs are variable and include dyspnea, increased respiratory sounds, visual impairment, draining skin lesions, and weight loss (2). Inflammatory ocular lesions or neuro–ophthalmic abnormalities such as Horner's syndrome, anisocoria, or an absent menace are common (7, 9, 12). The involvement of the central nervous system has been reported in 30–41% of cats and results in ataxia, paresis, circling and head tilt (6, 7, 9). Even though uncommon, CNS infection may occur in isolation without active systemic disease (13).

Computed tomography (CT) and magnetic resonance imaging (MRI) findings in dogs with intracranial blastomycosis are variable and include solitary or multifocal mass lesions that can be intra- and/or extra-axial and may extend from the nasal cavity or orbit into the cranial vault, perilesional edema, mass–effect and brain herniation, meningeal thickening and contrast enhancement, and ventricular lesions characterized by ventriculomegaly, ependymal or periventricular contrast enhancement, and evidence of increased intracranial pressure (14–19). Reports of imaging findings in cats with intracranial blastomycosis are limited. The CT findings in one domestic cat included a large peripheral intra-cranial strongly contrast-enhancing mass with associated meningeal enhancement, perilesional edema, mass-effect, and associated midline shift (13). The MRI findings reported in a tiger (*Panthera tigris*) included asymmetric hydrocephalus with marked contrast enhancement of the ventricular lining (20).

In this case series, we report the MRI findings in three domestic cats diagnosed with CNS blastomycosis. Case 2 was included in a previous publication on ocular blastomycosis (12), but diagnostic imaging findings were not included in the prior study and are presented here.

The MRI scans were performed using a 1.0 T MRI system (MAGNETOM Harmony™, Siemens Medical Solutions, Malvern, PA) (Case 1 first scan and Case 2) or a 1.5T MRI system (MAGNETOM Espree™, Siemens Medical Solutions, Malvern, PA) (Case 1 second scan and Case 3). Intravenous contrast medium was administered in all cases (Cases 1 and 2: 0.1 mmol/kg IV gadopentetate meglumine, Magnevist^®^, Bayer Schering Pharma; Case 3: 0.1 mmol/kg IV gadodiamide, Omniscan^®^, GE Healthcare). The MRI protocols varied slightly between the cases. The sequences acquired in all patients included sagittal T2-weighted spin echo (SE); transverse T2-weighted SE, T1-weighted SE, proton-density (PD)-weighted SE, T2-FLAIR, and T2^*^-weighted gradient recalled echo (GRE); transverse postcontrast volume interpolated GRE images with fat saturation (FatSat); and postcontrast transverse, dorsal FatSat and sagittal T1-weighted SE images. Diffusion-weighted imaging (DWI) and apparent diffusion coefficient (ADC) maps were available for review for the second scan in Case 1 and Case 3.

## Case descriptions

### Case 1

An 11-year-old male castrated 5.7-kg domestic shorthair cat presented to the internal medicine service after experiencing two seizures (believed to be tonic–clonic). The medical record information prior to this presentation is limited. The patient was feline immunodeficiency virus (FIV) positive and had been seen at the referring veterinarian 3 months prior with a presenting complaint of fever and ataxia. Pulmonary nodules were identified on thoracic radiographs at that time. A diagnosis of systemic blastomycosis was made based on fine-needle aspiration (FNA) of an enlarged popliteal lymph node. A *Blastomyces* urine antigen enzyme immunoassay (*Blastomyces* quantitative antigen EIA; MiraVista Diagnostics; range of quantification 0.2–14.7 ng/ml) was performed and was highly positive (above the limit of quantification). The clinical signs and lung nodules had improved with treatment (itraconazole and amphotericin B; dose regimen unknown). The reported tonic–clonic seizures occurred after the most recent antifungal treatment had been administered. At presentation, no abnormalities were noted on physical and neurologic examination.

**Figure 1:**
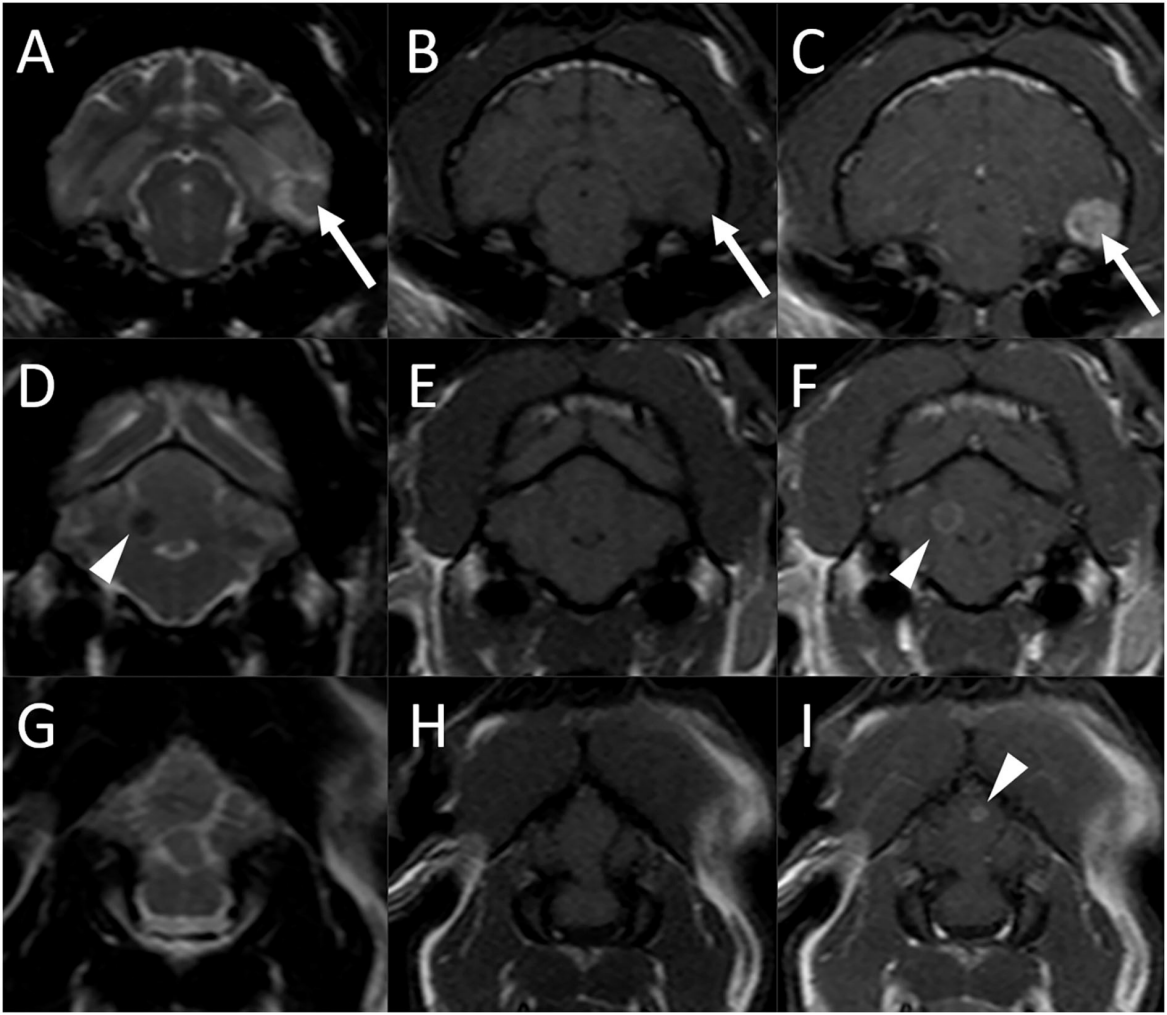
Brain MRI in an 11-year-old male castrated domestic shorthair cat (Case 1) 3 months after the diagnosis of systemic blastomycosis and after experiencing two seizures after the latest antifungal treatment. Transverse T2-weighted **(A)**, **(D)**, and **(G)**, T1-weighted **(B)**, **(E)**, and **(H)**, and postcontrast T1-weighted **(C)**, **(F)**, and **(I)** images. **(A)–(C)** A round ≈0.8-cm diameter nodule is associated with the ventral aspect of the left temporal lobe (arrows) which is iso-to slightly hypointense to gray matter, with a hyperintense rim on T2-weighted image **(A)**, slightly hypointense on T1-weighted image **(B)**, and has strong and homogeneous contrast enhancement **(C)**. There is moderate indistinct perilesional T2 hyperintensity, consistent with perilesional edema. **(D)–(E)** A second well-circumscribed ≈0.4-cm diameter T2 hypointense **(A)** and T1 isointense **(B)** lesion is associated with the right cerebellar hemisphere and exhibits moderate ring enhancement **(C)** following contrast medium administration (arrowheads). **(G)–(I)** A third ≈0.2-cm diameter ring-enhancing lesion is noted in the cerebellar vermis [**(I)**, arrowhead], not seen on the precontrast images.

On MRI of the brain, three separate intra-axial lesions were identified (Figure 1). A round ≈0.8-cm diameter nodule was associated with the ventral aspect of the left temporal lobe. This lesion was iso-to-slightly hypointense to gray matter, with a hyperintense rim on T2-weighted and T2-FLAIR images, was slightly hypointense on T1-weighted images, did not show evidence of susceptibility artifact on T2^*^-weighted images, and was strongly homogeneously contrast enhancing. There was moderate indistinct perilesional T2 hyperintensity, consistent with perilesional edema. A second well-circumscribed ≈0.4-cm diameter T2 hypointense and T1 isointense lesion was associated with the right cerebellar hemisphere and exhibited moderate ring enhancement following contrast medium administration. A third ≈0.2-cm diameter ring-enhancing lesion was noted in the cerebellar vermis, not seen on precontrast images. There was no evidence of perilesional edema associated with the cerebellar lesions. There was no evidence of meningeal enhancement or any abnormalities of the ventricular system, skull, nasal cavity, paranasal sinuses, tympanic bullae, orbits, ocular structures, and soft tissues of the head and cranial neck including regional lymph nodes.

A cisternal cerebrospinal fluid (CSF) tap was obtained and yielded a diagnosis of probably iatrogenic hemorrhage without other cytologic abnormalities. A *Blastomyces* urine antigen EIA was moderately positive (3.04 ng/ml).

Corticosteroid treatment was initiated (prednisolone 5-mg/head PO q 24 h × 3 days, 2.5-mg/head PO q 24 h × 3 days, and 1.25-mg/head PO q 24 h × 3 days) for treatment of perilesional edema and to help manage the inflammatory effects caused by fungal die-off. The patient was also started on phenobarbital (2.5-mg/kg PO BID of 20-mg/ml suspension), levetiracetam (20-mg/kg PO of 100-mg/ml suspension q 8 h), and fluconazole (50-mg/head PO q 12 h). Amphotericin B was discontinued. Phenobarbital was discontinued the following day due to the patient being very lethargic.

The cat showed improvement over the next months and was considered clinically normal aside from an occasional cough and unrelated chronic gingivitis 2 months after the MRI examination (5 months after initial diagnosis). The previously seen pulmonary abnormalities had resolved. There was no recurrence of seizures, and no other neurologic abnormalities were reported. A *Blastomyces* urine antigen EIA was greatly improved but still mildly positive (0.73 ng/ml).

The patient remained neurologically normal. Levetiracetam was gradually tapered from 5 to 8 months after the initial diagnosis without recurrence of seizure activity. A *Blastomyces* urine antigen EIA was still mildly positive after 7 and 9 months. Fluconazole was discontinued 15 months after the initial diagnosis (12 months after the first MRI examination) based on 2 consecutive negative *Blastomyces* urine antigen EIA tests.

The cat represented 3 years later (4 years and 7 months after initial diagnosis) to the emergency service for a 24-h history of circling to the right and behavior changes. On neurologic examination, the patient was circling to the right and had a bilaterally absent menace response. Possible hyperesthesia of the head was also noted. Neurolocalization was to the right forebrain. Thoracic radiographs did not reveal any significant abnormalities.

**Figure 2:**
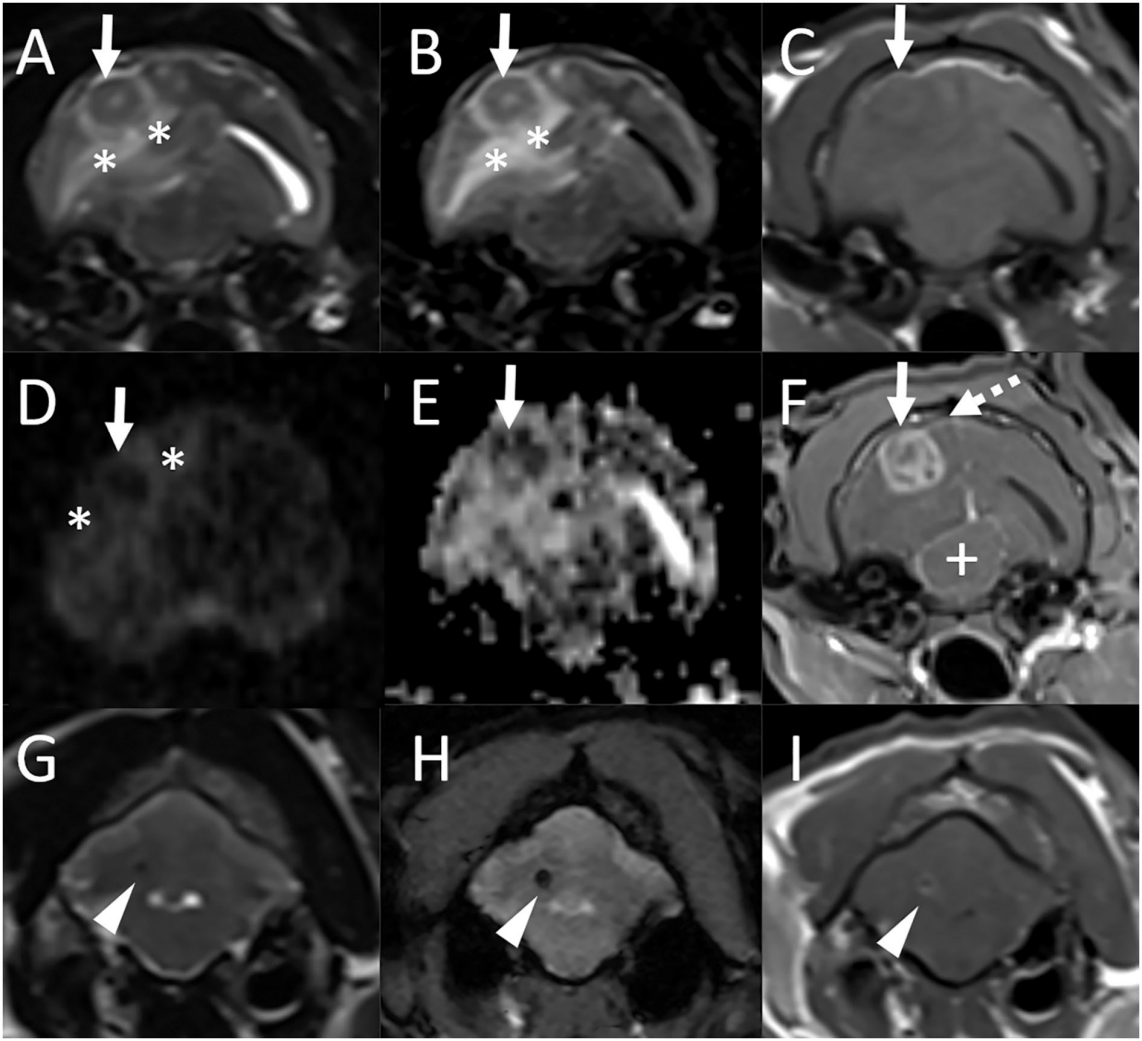
Repeat MRI examination of the cat in Figure 1 (Case 1), 4 years and 4 months after the initial MRI study. Transverse T2-weighted **(A)**, T2-FLAIR **(B)**, T1-weighted **(C)**, diffusion-weighted imaging [DWI, **(D)**] and corresponding apparent diffusion coefficient (ADC) map **(E)**, and postcontrast T1-weighted GRE with fat suppression **(F)** images at the level of the tympanic bullae and temporal lobes, and transverse T2-weighted **(G)**, T2*-weighted GRE **(H)**, and postcontrast T1-weighted **(I)** images at the level of the cerebellum. **(A)–(F)** There is a new finding of an ≈1.2 × 1.4 cm ovoid mass in the right temporal lobe (solid arrows) which is predominantly T2, T2-FLAIR, and T1 isointense to gray matter with a small centrally located T2 hyperintense and T1 hypointense focus which does not suppress on T2-FLAIR [**(A)–(C)**]. The lesion is hypointense both on DWI and ADC maps [**(D)**, **(E)**]. There is marked perilesional T2 and FLAIR hyperintensity following the white matter tracts of the right cerebral hemisphere (*) with a moderate to marked mass effect characterized by compression of the right lateral ventricle, leftward midline shift, and compression of the mesencephalon (+). Following contrast medium administration, the mass displays marked heterogeneous contrast enhancement **(F)**. Mild focal pachymeningeal contrast enhancement is identified adjacent to the lesion (dashed arrow). An incidental small amount of fluid is noted within the bilateral tympanic bulla. **(G)–(I)** An ≈0.2-cm diameter well-defined T2 hypointense ovoid nodule is observed in the region of the previously seen cerebellar hemispheric lesion and is decreased in size from previous [**(G)**, arrowhead]. This lesion is associated with a susceptibility artifact on T2*-weighted images **(H)** and has mild rim contrast enhancement **(I)**.

Magnetic resonance imaging was repeated. There was a new finding of an ≈1.2 × 1.4 cm ovoid mass in the right temporal lobe (Figure 2) which was predominantly T2 and T1 isointense to gray matter with a small centrally located T2 hyperintense and T1 hypointense focus which did not suppress on T2-FLAIR. The lesion was hypointense on both DWI and ADC maps. There was marked perilesional T2 and FLAIR hyperintensity following the white matter tracts of the right cerebral hemisphere with a moderate to marked mass effect characterized by compression of the right lateral ventricle, leftward midline shift, and caudal transtentorial and foramen magnum herniations. Following contrast medium administration, the mass displayed marked heterogeneous contrast enhancement. Mild focal pachymeningeal contrast enhancement was identified adjacent to the lesion. An ≈0.2-cm diameter well-defined T1, T2, and T2-FLAIR hypointense ovoid nodule was observed in the region of the previously seen cerebellar hemispheric lesion and was decreased in size. This lesion was associated with a susceptibility artifact on T2^*^-weighted images and had mild rim contrast enhancement. The lesion within the cerebellar vermis was no longer visible. An incidental small amount of fluid was noted within the bilateral tympanic bulla. The remaining structures of the head and cranial neck remained normal.

The patient received a tapering dosing regimen of prednisolone (5-mg/head PO q 24 h × 3 days, 2.5-mg/head PO q 24 h × 3 days, and 1.25-mg/head PO q 24 h × 3 days). Fluconazole treatment was resumed empirically (50-mg/head PO q 12 h) while awaiting urine antigen testing results. A repeat *Blastomyces* urine EIA test was positive (4.24 ng/ml), and fluconazole treatment was continued. The patient improved, and the client reported the cat as being back to normal 5 weeks after the second MRI examination. On neurologic examination at that time, menace response was noted in both eyes. The pupillary light responses were decreased. Additional findings during this visit included weight loss, hypoalbuminemia, anemia, a soft heart murmur, and an irregular heartbeat. The patient represented for a work-up of these new problems to the internal medicine service 2 weeks later and at that point was diagnosed with large cell gastrointestinal (GI) lymphoma. Chemotherapy was started and initially well-tolerated. The owners did not report a recurrence of neurologic abnormalities, and the physical examinations performed by the oncology service over the next weeks did not reveal any obvious neurologic abnormalities. A full neurologic exam was not performed. Urine blastomycosis antigen tests were also not repeated during that time due to the inability to acquire sufficient urine samples. The patient started declining ≈2 months after the diagnosis of GI lymphoma and was euthanized 3 months later. An autopsy was not performed. The overall survival time after the initial diagnosis of systemic blastomycosis was ≈5 years.

### Case 2

**Figure 3:**
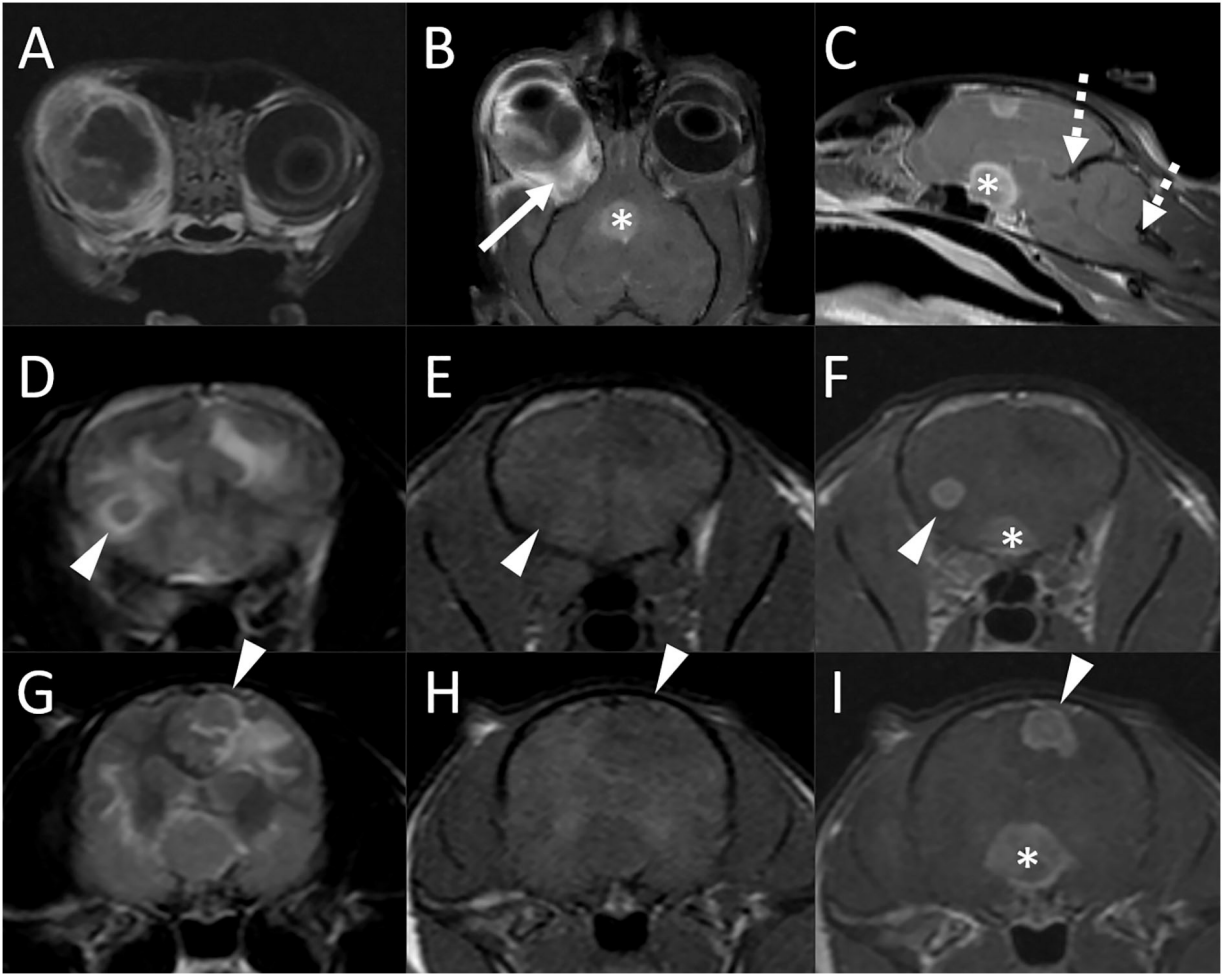
Brain MRI in a 3-year-old domestic shorthair cat (Case 2) following a diagnosis of ocular blastomycosis and with concern for intracranial involvement. Transverse **(A)**, dorsal **(B)**, and midline sagittal **(C)** postcontrast T1-weighted images; transverse T2-weighted images [**(D)** and **(G)**], transverse T1-weighted images before [**(E)** and **(H)**] and after [**(F)** and **(I)**] contrast medium administration. There is severe thickening, diffuse heterogeneous alteration in signal intensity, abnormal contrast enhancement of the periocular and tissues, thickening and enhancement of the wall of the globe, and retinal detachment [**(A)** and **(B)**]. There is diffuse thickening of the right optic nerve [**(B)**, arrow] and marked enlargement of the optic chiasm [* in **(B)**, **(C)**, **(F)**, and **(I)**]. A round 0.5-cm diameter intra-axial nodule is associated with the caudal aspect of the right frontal lobe [arrowheads, **(D)–(F)**] and an irregularly shaped ≈0.8 cm × 0.7 cm nodule is associated with the left parietal lobe [**(G)–(I)**, arrowheads]. Both lesions are iso- to mildly hypointense to gray matter on both T2 and T1-weighted images [**(D)**, **(E)**, **(G)**, **(H)**]. Bordering the lesions and extending extensively throughout the cerebral white matter bilaterally, there are parenchymal hyperintensities consistent with vasogenic edema. There is evidence of increased intracranial pressure characterized by attenuation of the CSF signal in the cerebral sulci, compression of the lateral ventricles, a mild midline shift, and caudal transtentorial and foramen magnum herniations [**(C)**, dashed arrows].

A 3-year-old 4.7-kg female spayed domestic shorthair cat presented to the emergency service after a mass was noted associated with the conjunctiva of her right eye. Two weeks prior she had been seen by the ophthalmology service for uveitis and secondary glaucoma of the right eye (Oculus Dexter; OD). Physical examination findings and a complete blood count (CBC) performed at that point were normal. The infectious disease testing for feline leukemia virus (FeLV), FIV, and toxoplasmosis were negative. The patient was managed with topical medications. At the current presentation, the cat had worsened uveitis OD and blindness in both eyes (Oculus Uterque; OU) with a fixed dilated pupil of the left eye (Oculus Sinister; OS). Thoracic radiographs revealed a single ≈2.5-cm diameter ill-defined soft tissue opacity mass associated with the left caudal lung lobe. A fine-needle aspiration of the conjunctival mass was performed and yielded a diagnosis of blastomycosis. Due to concern for intracranial involvement, MRI of the brain was performed (Figure 3).

There were marked abnormalities of the right eye including severe thickening and contrast enhancement of the periocular tissues, thickening and enhancement of the wall of the globe, retinal detachment, diffuse heterogeneous alteration in signal intensity of the ocular structures with abnormal contrast enhancement, and multifocal susceptibility artifacts, mostly in the posterior chamber, consistent with hemorrhage. There was diffuse thickening of the right optic nerve (up to 5 mm) and marked enlargement of the optic chiasm (1.3 cm). Two additional intracranial lesions were noted. A round 0.5 cm diameter intra-axial nodule was associated with the caudal aspect of the right frontal lobe. An irregularly shaped ≈0.8 × 0.7 cm nodule was associated with the left parietal lobe. This lesion was in contact with the falx and meningeal surface of the brain but formed an acute angle with both. Both lesions were iso- to mildly hypointense to gray matter on both T1- and T2-weighted images. Bordering the lesions and extending extensively throughout the cerebral white matter bilaterally, there were parenchymal hyperintensities consistent with vasogenic edema. There was evidence of increased intracranial pressure characterized by attenuation of the CSF signal in the cerebral sulci, compression of the lateral ventricles, a mild midline shift, and caudal transtentorial and foramen magnum herniations. Following contrast medium administration, the right globe, abnormal tissues within the right orbit, right optic nerve, optic chiasm, and intracranial nodules showed strong and homogeneous contrast enhancement. There was no evidence of abnormal meningeal contrast enhancement and no evidence of a dural tail sign adjacent to the brain lesions. Moderate enlargement of the right and mild enlargement of the left medial retropharyngeal lymph nodes were noted.

The patient was euthanized, and an autopsy was performed, confirming the clinical diagnosis of blastomycosis. There was marked pyogranulomatous panophthalmitis of the right eye with involvement of the optic nerve and the optic chiasm. Both cerebral nodules were intra-axial in location, and a diagnosis of a marked pyogranulomatous meningoencephalitis with intralesional yeasts was made. Additional lesions were found in the lungs (as suspected based on radiographs), the thoracic lymph nodes, and the fourth and fifth digits of the right front limb.

### Case 3

An estimated 7-year-old 6.1-kg male castrated domestic shorthair cat presented to a local emergency clinic for head pressing. Complete blood count and blood chemistry panel were normal, and FeLV/FIV testing was negative. The patient was referred for progressive neurologic abnormalities. Upon presentation, the cat was bright, alert, and responsive. One hind limb had been amputated previously for unknown reasons. Neurological deficits included a lack of menace response of the left eye (OS) and intermittent loss of the right eye (OD), bilateral (OU) loss of direct and indirect pupillary light reflexes, and circling to the right. Neurolocalization was to the right forebrain and bilateral optic nerves/optic chiasm. A mild diffuse bronchointerstitial pattern was identified on thoracic radiographs.

**Figure 4:**
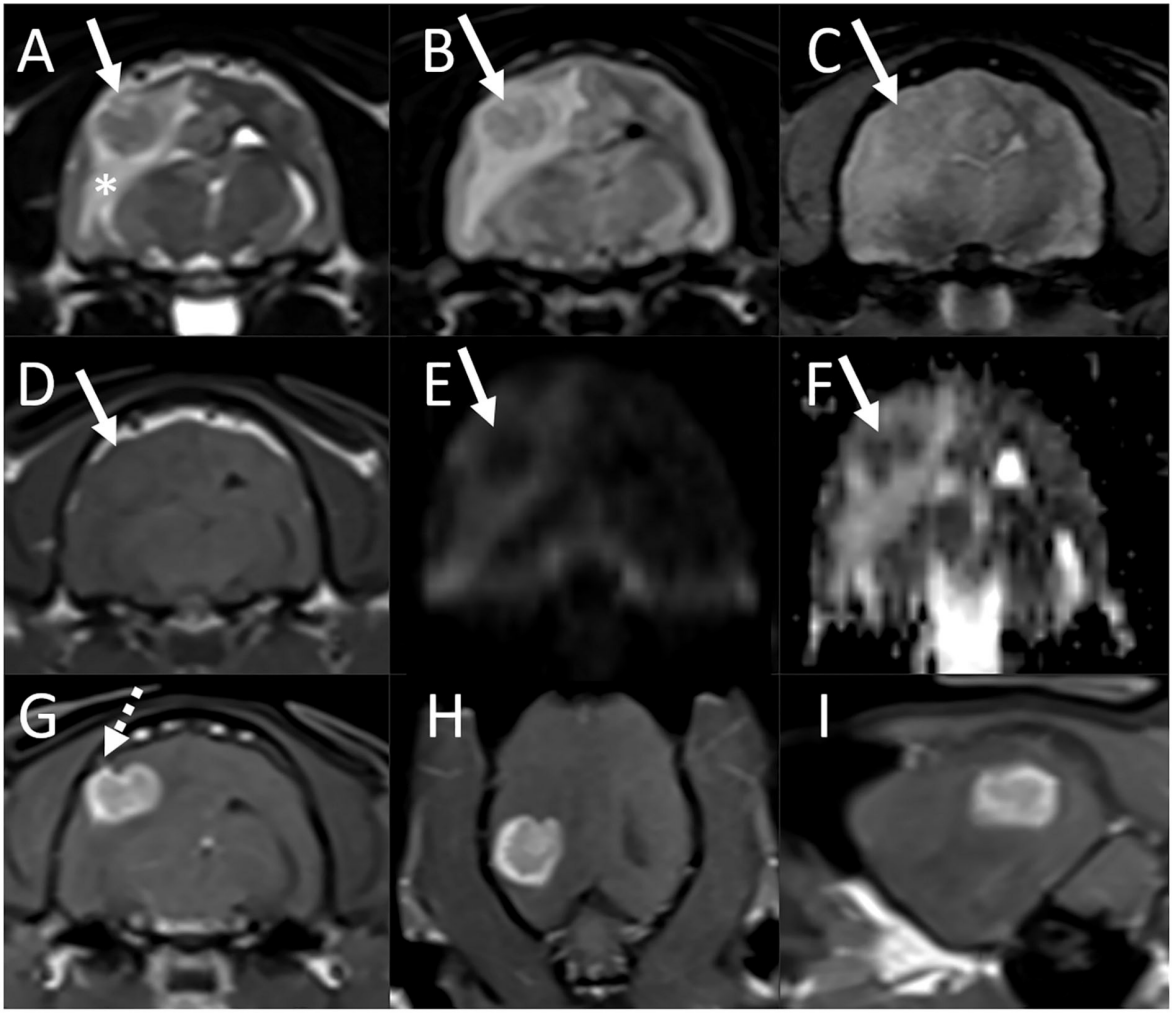
Brain MRI in an estimated 7-year-old cat (Case 3) with presenting complaints of head pressing and progressive neurologic deficits. Transverse T2-weighted **(A)**, T2-FLAIR **(B)**, T2*-weighted GRE **(C)**, T1-weighted **(D)**, diffusion-weighted imaging [DWI, **(E)**] and corresponding ADC map **(F)**, and postcontrast T1-weighted images in the transverse **(G)**, dorsal **(H)**, and sagittal **(I)** planes. **(A)–(F)** There is an ≈1-cm diameter mass associated with the periphery of the right temporal lobe, in contact with the dural surface (arrows). The lesion is T2, T2-FLAIR, and T1 iso- to mildly hypointense to gray matter [**(A)**, **(B)**, **(D)**], is not associated with susceptibility artifact on T2*-weighted image **(C)**, and is hypointense both on DWI and ADC maps [**(E)** and **(F)**]. There is marked perilesional T2 hyperintensity extending along the white matter tracts of the right cerebral hemisphere, consistent with vasogenic edema, resulting in compression of the right lateral ventricle, and a midline shift toward the left. A small volume of fluid is present in the nasopharynx, considered incidental. **(G)–(I)** Following contrast medium administration, the mass shows strong and fairly homogeneous contrast enhancement. The majority of the lesion is centered on brain parenchyma, with acute angles between the ventral, rostral, and caudal aspects of the mass and the calvarium. At the dorsal aspect of the mass, there is mild focal thickening and contrast enhancement of the pachymeninges [**(G)**, dashed arrow].

On MRI examination of the brain, there was an ≈1-cm diameter mass associated with the periphery of the right temporal lobe, in contact with the dural surface (Figure 4). The lesion was iso-to-mildly hypointense to gray matter on both T1- and T2-weighted images, was not associated with susceptibility artifact on T2^*^-weighted images, and was hypointense on both DWI and ADC maps. There was marked perilesional T2 hyperintensity extending along the white matter tracts of the right cerebral hemisphere, consistent with vasogenic edema, resulting in compression of the right lateral ventricle, a midline shift toward the left, caudal transtentorial, and mild foramen magnum herniations. Following contrast medium administration, the mass showed strong and fairly homogeneous contrast enhancement. The majority of the lesion was centered on brain parenchyma, with acute angles between the ventral, rostral, and caudal aspects of the mass and the calvarium. At the dorsal aspect of the mass and best seen on thin section postcontrast T1-weighted FatSat GRE images, there was mild focal thickening and contrast enhancement of the pachymeninges. A small volume of fluid was present in the nasopharynx, considered incidental. There were no abnormalities of the skull, nasal cavity, paranasal sinuses, tympanic bullae, orbits, ocular structures, and soft tissues of the head and cranial neck including lymph nodes.

A CSF tap was not performed. The patient was administered mannitol (0.5 g/kg IV) and dexamethasone sodium phosphate (0.1 mg/kg IV). Blood and urine samples were submitted for cryptococcosis and blastomycosis testing, respectively. The patient was discharged the following day on prednisolone (2-mg/head PO q 24 h) and levetiracetam (20-mg/kg PO of 100 mg/ml suspension q 8 h).

A cryptococcus antigen latex test was negative. A *Blastomyces* urine antigen EIA was positive (6.11 ng/ml). The patient was prescribed fluconazole (50-mg/head PO q 24 h). Two days after the start of treatment, the owner reported sudden deterioration of the patient followed by death. An autopsy was not performed.

## Discussion

Inflammatory and neoplastic etiologies account for the majority of brain diseases in cats (21, 22), and an imaging diagnosis may not always be straightforward. This study aims at describing imaging features in cats with blastomycosis, an uncommon cause of meningoencephalitis in this species.

Even though the imaging findings in the three cats included in this case series differed, some commonalities were observed. All animals had focal or multifocal mass lesions associated with the forebrain. Contact with the brain surface was common, resulting in difficulty classifying a lesion as intra-axial vs. extra-axial in some instances. Ultimately, intra-axial lesion location was considered most likely in all cats based on lesion centering on brain parenchyma, infiltrative rather than expansile growth of the mass, the presence of additional intra-axial lesions in the majority of cases including cerebellar lesions in one cat, and the acute rather than the obtuse angle formed between the periphery mass and the skull (comparable to the radiographic differentiation of a pulmonary vs. an extrapleural mass based on contact with the thoracic wall on radiographs). An autopsy was only performed on one cat, confirming the intra-axial location of the lesion. Difficulty in distinguishing intra- from extra-axial lesion location was mentioned in a previous study describing CT findings in a cat with an intracranial blastomycosis granuloma (13) and has also been described in dogs and cats with CNS coccidiomycosis (23). Dural contact has been described as an MRI finding that may help increase the index of suspicion for intracranial granuloma over glioma in dogs (19).

In the acute stage, brain lesions were iso- to mildly hypointense to gray matter on T2-weighted images, variable but generally isointense on T1-weighted images, and strongly contrast-enhancing. The T2 hypointensity has previously been reported with fungal granulomas (17, 19, 23, 24). This may be an important consideration when prioritizing differential diagnoses considering the myriad possibilities for single or multifocal intra-axial mass(es). Gliomas are intra-axial tumors that are rare in cats and are reported to be T2 hyperintense and characterized by rim rather than homogeneous contrast enhancement (25). Intralesional T2 hypointensity may be seen with meningiomas if mineralization or hemorrhage is present (25, 26). These tumors are also commonly strongly and homogeneously contrast-enhancing. However, the T2 hypointensities are typically foci within an overall iso- to hyperintense mass, unlike the Blastomyces granulomas described here which were more uniformly T2 hypointense. In addition, meningiomas are extra-axial and characterized by broad-based or plaque-like contact with the skull. Intracranial melanoma metastases or hemorrhagic lesions (benign or malignant) may be T2 hypointense (27). However, those types of lesions are rare in cats and will typically exhibit marked susceptibility artifacts on T2^*^-weighted GRE images which were not seen in the cases described here. CNS lymphoma has a variable imaging appearance in cats, with intra- and/or extra-axial masses of varying intensity and contrast enhancement patterns. While T2 hyperintensity is a more common feature and T2 hypointensity has not been reported, T2 isointensity is possible (28). Other substances and lesions that may appear hypointense on T2-weighted images are gadolinium-based contrast agents, mucous- or protein-containing lesions, highly cellular lesions, lesions containing mineral substances such as calcium, copper, or iron, turbulent and rapid blood (or CSF) flow, and air-containing spaces or lesions (29). The T2 hypointensity seen with fungal granulomas can be explained by their high cellularity and density limiting the mobility of intralesional hydrogen protons. Other contributing factors (such as the presence of protein or mineralization) are possible but are not supported by the lesion appearance on other sequences.

Other common imaging features of acute granulomas in this case series were strong contrast enhancement of the main lesion, meningeal enhancement, perilesional edema, and mass effect. This is similar to the findings of previous reports (13, 14, 16–19). Vasogenic edema in Case 1 at first presentation was attributed to a flare-up of a granuloma following antifungal treatment. Chronic granulomas otherwise did not have evidence of perilesional edema, similar to previous reports (17), and were ring-enhancing. The extension of ocular blastomycosis into the cranial vault to affect the optic chiasm in one cat was similar to a case of intracranial extension of a retrobulbar fungal granuloma reported in a dog (14). The appearance of the fungal granulomas in two cases where DWI was performed was interesting. A restricted diffusion (characterized by hyperintensity on DWI and hypointensity on the ADC map) has been reported in some intracranial fungal infections in people (30). The hypointense appearance of the granulomas on both DWI and ADC in our cases is most likely attributable to “T2 blackout effects,” where the true diffusion signal is reduced on DWI images by the presence of a very low signal in a lesion. This has been reported in acute and early subacute intracerebral hematomas in people (31). Interestingly, neither of our cases exhibiting this imaging feature had evidence of susceptibility artifact in the lesion indicative of hemorrhage. It appears that the T2 hypointense nature of the granuloma was accentuated by surrounding vasogenic edema which was most evident on DWI/ADC.

Although there are a variety of imaging descriptions of intracranial blastomycosis in dogs, it is worth mentioning one particular manifestation centered on the ventricular system and resulting in periventricular edema, periventricular and meningeal contrast enhancement, and ventriculomegaly (15). Although this appearance has not been described in domestic cats with CNS blastomycosis, similar MR abnormalities have been reported in a tiger (20). It warrants particular discussion in regards to feline patients, as this imaging manifestation would be similar to and possibly indistinguishable from feline infectious peritonitis (FIP) (32). The presence and type of concurrent systemic disease, FNA and cytology of lesions, CSF abnormalities, and fungal antigen testing would all be useful in differentiating between systemic fungal disease and FIP in a given feline patient.

The presence or absence of additional lesions outside the CNS is an important consideration when ranking differential diagnoses for intracranial lesions and prognostication. Most animals with CNS blastomycosis have systemic disease. Two of the cats in this study had evidence of single or multiple pulmonary masses. It is unclear if the mild bronchointerstitial pattern seen in the third patient was related or unrelated to fungal disease. Even though the prognosis for dogs and cats with blastomycosis is generally good, the two prognostic factors for survival are the severity of lung disease and CNS involvement (2). The long-term treatment and survival of one immunocompromised cat with both pulmonary and CNS blastomycosis is somewhat surprising but supports the suggestion that prolonged duration of treatment is indicated in patients with fungal encephalitis (33).

There were several limitations to this study. The case number is small, which is likely due to the relative rarity of blastomycosis in cats. In addition, it is likely that many animals with systemic disease present with other clinical signs and may not undergo brain MRI even if neurologic abnormalities are present. The diagnosis of blastomycosis in two cats was based on cytology, and additional testing (mycological isolation or polymerase chain reaction) was not performed. This is representative of clinical veterinary practice, where organism identification through cytologic or histopathologic evaluation is preferred over serology due to the associated cost for the client (2). The identification of medium-sized, spherical, 8–20 μm in diameter, deeply basophilic organisms with a refractile double wall and occasional broad-based budding suggests blastomycosis (2, 34). Fungal serology is typically recommended when direct visualization of the organism is not possible (24). Most cases of blastomycosis in humans, dogs, and cats are thought to be caused by *B. dermatitidis*; however, in 2013, *B. gilchristii*, which is genetically distinct, was reported to also be a possible cause (35, 36). Because of morphological and clinical similarities between *B. dermatitidis* and *B. gilchristii*, there is no clinical need for genetic sequencing to differentiate the two species (12). In people, atypical blastomycosis is occasionally caused by *Blastomyces helicus* in western parts of North America and *Blastomyces percursus* in Africa (36). Neither of these organisms has been reported in small animals in the Southeastern United States to date. For these reasons, and considering the geographic distribution of various species of Blastomyces, *B. dermatitidis* was considered the most likely infectious agent seen in our case population. The diagnosis in one case was based on a positive result for a *Blastomyces* urine antigen EIA. This assay is only labeled for use in dogs, and it is known to cross-react with *Histoplasma capsulatum* antigen as well as possibly other infectious agents (*Paracoccidioidomyces, Penicillium*, less frequently in *Coccidiooidomyces*, rarely *Aspergillus*, and possibly *Sporotrichium*) (37, 38). However, most of these are not endemic in Tennessee, many have not been reported in cats, and CNS involvement in cats has to our knowledge not been reported in any of them (39, 40). Intracranial blastomycosis, therefore, remains the most likely diagnosis in our patient. Finally, the MRI protocols were not standardized, and DWI/ADC images were only available for review in two scans. Despite these limitations, this study provides important information on MRI findings in cats with CNS blastomycosis.

In summary, the imaging features that may suggest blastomycosis over other intracranial diseases in cats include focal or multifocal intra-axial mass lesions with dural contact, hypointensity on T2-weighted images and DWI/ADC, strong and homogeneous contrast enhancement of the lesion(s), concurrent meningeal enhancement, marked perilesional edema and mass-effect, and ocular abnormalities. Future studies with larger case numbers are warranted to confirm these findings and identify possible other imaging manifestations of CNS blastomycosis in feline patients.

## Data availability statement

The original contributions presented in the study are included in the article/supplementary material, further inquiries can be directed to the corresponding author/s.

## Ethics statement

Ethical review and approval was not required for the animal study because it is not required for retrospective case reports. Written informed consent was obtained from owners for participation of their animals.

## Author contributions

SH identified the cases to be included in the study, performed the image evaluation, and drafted the initial manuscript. JM and HS assisted with the medical record review of the patients and reviewed and revised the manuscript. All authors contributed to the manuscript's final revision and approved the submitted manuscript.

## Conflict of interest

The authors declare that the research was conducted in the absence of any commercial or financial relationships that could be construed as a potential conflict of interest.

## Publisher's note

All claims expressed in this article are solely those of the authors and do not necessarily represent those of their affiliated organizations, or those of the publisher, the editors and the reviewers. Any product that may be evaluated in this article, or claim that may be made by its manufacturer, is not guaranteed or endorsed by the publisher.

## References

[1] Gnat S Lagowski D Nowakiewicz A Dylag M. A global view on fungal infections in humans and animals: infections caused by dimorphic fungi and dermatophytoses. J Appl Microbiol. (2021) 131:2688–704. 10.1111/jam.15084 33754409

[2] Legendre AM. Blastomycosis. In: Greene CE editor. Infectious Diseases of the Dog and Cat. 4th edn. St. Louis: Elsevier Saunders (2012). p. 606–13.

[3] Schwartz IS. Blastomycosis in mammals. In: Seyedmousavi S de Hoog G Guillot J Verweij P editors. Emerging and Epizootic Fungal Infections in Animals. Cham: Springer International Publishing AG, part of Springer Nature (2018). p. 159–76. 10.1007/978-3-319-72093-7_8

[4] Arceneaux KA Taboada J Hosgood G. Blastomycosis in dogs: 115 cases (1980–1995). J Am Vet Med Assoc. 1998;213:658. 9731260

[5] Breider MA Walker TL Legendre AM Vanee RT. Blastomycosis in cats – 5 cases (1979–1986). J Am Vet Med Assoc. (1988) 193:570–2. 3170335

[6] Davies C Troy GC. Deep mycotic infections in cats. J Am Anim Hosp Assoc. (1996) 32:380–91. 10.5326/15473317-32-5-380 8875352

[7] Gilor C Graves TK Barger AM O'Dell-Anderson K. Clinical aspects of natural infection with Blastomyces dermatitidis in cats: 8 cases (1991–2005). JAVMA-J Am Vet Med A. (2006) 229:96–9. 10.2460/javma.229.1.96 16817721

[8] Legendre AM Walker M Buyukmihci N Stevens R. Canine Blastomycosis – a review of 47 clinical cases. J Am Vet Med Assoc. (1981) 178:1163–8. 7275754

[9] Miller PE Miller LM Schoster JV. Feline Blastomycosis – a report of 3 cases and literature-review (1961 to 1988). J Am Anim Hosp Assoc. (1990) 26:417–24.

[10] Davies JL Epp T Burgess HJ. Prevalence and geographic distribution of canine and feline blastomycosis in the Canadian prairies. Can Vet J-Revue Veterinaire Canadienne. (2013) 54:753–60. 24155475 PMC3711163

[11] Lloret A Hartmann K Pennisi MG Ferrer L Addie D Belak S. Rare systemic mycoses in cats: blastomycosis, histoplasmosis and coccidioidomycosis: ABCD guidelines on prevention and management. J Feline Med Surg. (2013) 15:624–7. 10.1177/1098612X13489226 23813828 PMC11148945

[12] Morris JM Sigmund AB Ward DA Hendrix DVH. Ocular findings in cats with blastomycosis: 19 cases (1978-2019). JAVMA-J Am Vet Med A. (2022) 260:422–7. 10.2460/javma.21.03.0135 34936573

[13] Smith JR Legendre AM Thomas WB LeBlanc CJ Lamkin C Avenell JS. Cerebral Blastomyces dermatitidis infection in a cat. JAVMA-J Am Vet Med A. (2007) 231:1210–4. 10.2460/javma.231.8.1210 17937550

[14] Baron ML Hecht S Westermeyer HD Mankin JM Novak JM Donnell RL. Intracranial extension of retrobulbar blastomycosis (*Blastomyces dermatitidis*) in a dog. Vet Ophthalmol. (2011) 14:137–41. 10.1111/j.1463-5224.2010.00850.x 21366831

[15] Bentley RT Reese MJ Heng HG Lin TL Shimonohara N Fauber A. Ependymal and periventricular magnetic resonance imaging changes in four dogs with central nervous system blastomycosis. Vet Radiol Ultrasound. (2013) 54:489–96. 10.1111/vru.12049 23663013

[16] Hecht S Adams WH Smith JR Thomas WB. Clinical and imaging findings in five dogs with intracranial blastomycosis (*Blastomyces dermatiditis*). J Am Anim Hosp Assoc. (2011) 47:241–9. 10.5326/JAAHA-MS-5573 21673331

[17] Lipitz L Rylander H Forrest LJ Foy DS. Clinical and magnetic resonance imaging features of central nervous system blastomycosis in 4 dogs. J Vet Int Med. (2010) 24:1509–14. 10.1111/j.1939-1676.2010.0581.x 20738772

[18] Saito M Sharp NJH Munana K Troan BV Tokuriki M Thrall DE. findings of intracranial blastomycosis in a dog. Vet Radiol Ultrasound. (2002) 43:16–21. 10.1111/j.1740-8261.2002.tb00436.x 11871374

[19] Diangelo L Cohen-Gadol A Heng HG Miller MA Hague DW Rossmeisl JH. Glioma mimics: magnetic resonance imaging characteristics of granulomas in dogs. Front Vet Sci. (2019) 6:286. 10.3389/fvets.2019.00286 31555671 PMC6722480

[20] Hecht S Cushing AC Williams-Hagler DA Craig LE Thomas WB Anderson KM. Magnetic resonance imaging in 50 captive non-domestic felids-Technique and imaging diagnoses. Front Vet Sci. (2022) 9:827870. 10.3389/fvets.2022.827870 35211543 PMC8861525

[21] Bradshaw JM Pearson GR Gruffydd-Jones TJ. A retrospective study of 286 cases of neurological disorders of the cat. J Comp Pathol. (2004) 131:112–20. 10.1016/j.jcpa.2004.01.010 15276850 PMC7134559

[22] Nakamoto Y Uemura T Hasegawa H Nakamoto M Ozawa T. Feline neurological diseases in a veterinary neurology referral hospital population in Japan. J Vet Med Sci. (2019) 81:879–85. 10.1292/jvms.18-0447 31061248 PMC6612503

[23] Bentley RT Heng HG Thompson C Lee CS Kroll RA Roy ME. Magnetic resonance imaging features and outcome for solitary central nervous system coccidioides granulomas in 11 dogs and cats. Vet Radiol Ultrasound. (2015) 56:520–30. 10.1111/vru.12258 25857572

[24] Lavely J Lipsitz D. Fungal infections of the central nervous system in the dog and cat. Clin Tech Small an P. (2005) 20:212–9. 10.1053/j.ctsap.2005.07.001 16317910

[25] Troxel MT Vite CH Massicotte C McLear RC Van Winkle TJ Glass EN. Magnetic resonance imaging features of feline intracranial neoplasia: retrospective analysis of 46 cats. J Vet Intern Med. (2004) 18:176–89. 10.1111/j.1939-1676.2004.tb00158.x 15058768

[26] Martin-Vaquero P Da Costa RC Aeffner F Oglesbee MJ Echandi RL. Imaging diagnosis—Hemorrhagic meningioma. Vet Radiol Ultrasound. (2010) 51:165–7. 10.1111/j.1740-8261.2009.01645.x 20402404

[27] Orton T Gaillard F. Intracranial Metastatic Melanoma. Radiopaedia (2021). 10.53347/rID-4935 (accessed May 13, 2022).

[28] Durand A Keenihan E Schweizer D Maiolini A Guevar J Oevermann A. Clinical and magnetic resonance imaging features of lymphoma involving the nervous system in cats. J Vet Int Med. (2022) 36:679–93. 10.1111/jvim.16350 35048412 PMC8965233

[29] Zimny A Neska-Matuszewska M Bladowska J Sasiadek MJ. Intracranial lesions with low signal intensity on T2-weighted MR images – review of pathologies. Pol J Radiol. (2015) 80:40–50. 10.12659/PJR.892146 25628772 PMC4307690

[30] Starkey J Moritani T Kirby P. MRI of CNS fungal infections: review of aspergillosis to histoplasmosis and everything in between. Clin Neuroradiol. (2014) 24:217–30. 10.1007/s00062-014-0305-7 24870817

[31] Silvera S Oppenheim C Touze E Ducreux D Page P Domigo V. Spontaneous intracerebral hematoma on diffusion-weighted images: influence of T2-shine-through and T2-blackout effects. Am J Neuroradiol. (2005) 26:236–41. 15709118 PMC7974085

[32] Crawford AH Stoll AL Sanchez-Masian D Shea A Michaels J Fraser AR. Clinicopathologic features and magnetic resonance imaging findings in 24 cats with histopathologically confirmed neurologic feline infectious peritonitis. J Vet Int Med. (2017) 31:1477–86. 10.1111/jvim.14791 28833469 PMC5598904

[33] Bentley RT Taylor AR Thomovsky SA. Fungal infections of the central nervous system in small animals. Clinical features, diagnosis, and management. Vet Clin N Am-Small. (2018);48:63. 10.1016/j.cvsm.2017.08.010 28988704

[34] Lane LV Yang PJ Cowell RL. Selected infectious agents. In: Valenciano AC, editor. Cowell and Tyler's Diagnostic Cytology and Hematologyof the Dog and Cat. 5th edn. St. Louis, MO: Elsevier (2020). p. 44–64. 10.1016/B978-0-323-53314-0.00003-1

[35] Brown EM McTaggart LR Zhang SX Low DE Stevens DA Richardson SE. Phylogenetic analysis reveals a cryptic species Blastomyces gilchristii, sp. nov within the human pathogenic fungus Blastomyces dermatitidis. PLoS ONE. (2013) 8:e59237. 10.1371/journal.pone.0059237 23533607 PMC3606480

[36] Schwartz IS Kauffman CA. Blastomycosis. Semin Respir Crit Care Med. (2020) 41:31–41. 10.1055/s-0039-3400281 32000282

[37] Connolly P Hage CA Bariola JR Bensadoun E Rodgers M Bradsher RW. Blastomyces dermatitidis antigen detection by quantitative enzyme immunoassay. Clin Vaccine Immunol. (2012) 19:53–6. 10.1128/CVI.05248-11 22116687 PMC3255946

[38] MiraVistaLabs [cited 2022 July 1]. Available from: https://miravistalabs.com/medical-fungal-infection-testing/antigen-detection/blastomyces-dermatitidis-quantitative-eia-test/

[39] Aulakh HK Aulakh KS Troy GC. Feline histoplasmosis: A retrospective study of 22 cases (1986–2009). J Am Anim Hosp Assoc. (2012) 48:182–7. 10.5326/JAAHA-MS-5758 22474046

[40] Bromel C Sykes JE. Histoplasmosis in dogs and cats. Clin Tech Small an Pract. (2005) 20:227–32. 10.1053/j.ctsap.2005.07.003 16317912

